# (*E*)-3-Hydr­oxy-*N*′-(2-hydroxy­benzyl­idene)-2-naphthohydrazide

**DOI:** 10.1107/S1600536809053793

**Published:** 2009-12-24

**Authors:** Hassan Hosseini Monfared, Rahman Bikas, Peter Mayer

**Affiliations:** a Department of Chemistry, Zanjan University 45195-313, Zanjan, Islamic Republic of Iran; b Department of Chemistry and Biochemistry, Ludwig-Maximilians-Universität, Butenandtstrasse 5–13, 81377 München, Germany

## Abstract

The title compound, C_18_H_14_N_2_O_3_, is an aroylhydrazone with an approximately planar structure [dihedral angle of 15.27 (13)° between the benzene ring and the naphthyl ring system], stabilized by intra­molecular N—H⋯O and O—H⋯N hydrogen bonds. Inter­molecular O—H⋯O hydrogen bonds with the keto group as acceptor lead to strands along [100]. In terms of graph-set analysis, the descriptor on the unitary level is *C*_1_^1^(6)*S*(6)*S*(6).

## Related literature

For historical background to aroylhydrazones, see: Savanini *et al.* (2002 ▶); Craliz *et al.* (1955 ▶); Pickart *et al.* (1983 ▶); Offe *et al.* (1952 ▶); Arapov *et al.* (1987 ▶); Ranford *et al.* (1998 ▶). For related structures, see: Liu *et al.* (2004 ▶); Lei *et al.* (2008 ▶). For graph-set analysis of hydrogen-bond networks, see: Bernstein *et al.* (1995 ▶); Etter *et al.* (1990 ▶).
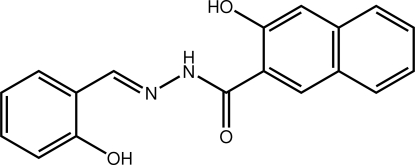


## Experimental

### 

#### Crystal data

C_18_H_14_N_2_O_3_
*M* *_r_* = 306.32
Orthorhombic, 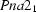

*a* = 12.6749 (4) Å
*b* = 4.9666 (1) Å
*c* = 22.7299 (6) Å
*V* = 1430.87 (7) Å^3^
*Z* = 4
Mo *K*α radiation
μ = 0.10 mm^−1^
*T* = 200 K
0.50 × 0.10 × 0.09 mm

#### Data collection

Nonius KappaCCD diffractometer
10328 measured reflections
1676 independent reflections
1416 reflections with *I* > 2σ(*I*)
*R*_int_ = 0.054

#### Refinement

*R*[*F* ^2^ > 2σ(*F* ^2^)] = 0.036
*wR*(*F* ^2^) = 0.095
*S* = 1.09
1676 reflections
220 parameters
1 restraint
H atoms treated by a mixture of independent and constrained refinement
Δρ_max_ = 0.16 e Å^−3^
Δρ_min_ = −0.17 e Å^−3^

### 

Data collection: *COLLECT* (Hooft, 2004 ▶); cell refinement: *SCALEPACK* (Otwinowski & Minor, 1997 ▶); data reduction: *DENZO* (Otwinowski & Minor, 1997 ▶) and *SCALEPACK*; program(s) used to solve structure: *SIR97* (Altomare *et al.*, 1999 ▶); program(s) used to refine structure: *SHELXL97* (Sheldrick, 2008 ▶); molecular graphics: *ORTEP-3* (Farrugia, 1997 ▶) and *Mercury* (Macrae *et al.*, 2006 ▶); software used to prepare material for publication: *PLATON* (Spek, 2009 ▶).

## Supplementary Material

Crystal structure: contains datablocks I, global. DOI: 10.1107/S1600536809053793/bg2308sup1.cif

Structure factors: contains datablocks I. DOI: 10.1107/S1600536809053793/bg2308Isup2.hkl

Additional supplementary materials:  crystallographic information; 3D view; checkCIF report

## Figures and Tables

**Table 1:** Hydrogen-bond geometry (Å, °)

*D*—H⋯*A*	*D*—H	H⋯*A*	*D*⋯*A*	*D*—H⋯*A*
O2—H2⋯N2	0.96 (5)	1.87 (5)	2.697 (3)	142 (4)
O3—H3⋯O1^i^	0.84 (4)	1.81 (4)	2.609 (2)	157 (3)
N1—H1⋯O3	0.92 (3)	1.85 (3)	2.628 (3)	141 (2)

Symmetry code: (i) 
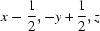
.

**Fig. 1.**
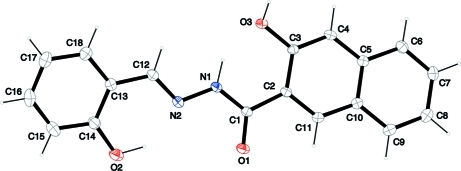
The molecular structure of the title compound, with atom labels and anisotropic displacement ellipsoids (drawn at 50% probability level) for non-H atoms.

**Fig. 2.**
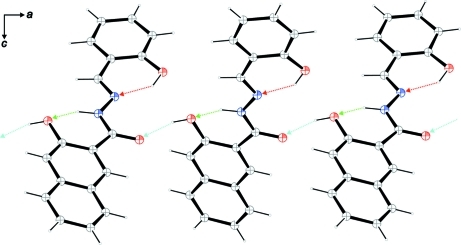
Hydrogen bonding in the title compound viewed along [0 - 1 0]. The green and red dashed arrows indicate intramolecular N–H···O and O–H···N hydrogen bonds, respectively. The blue dashed arrows indicate intermolecular O–H···O hydrogen bonds leading to strands along [100].

**Fig. 3.**
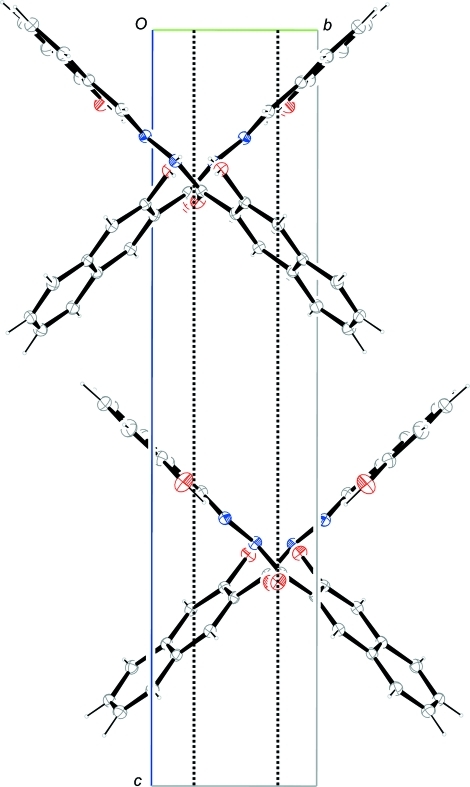
Projection of the hydrogen-bond linked chains viewed along [100]. For clarity only the two chains with their axis located within the unit cell are shown. Dashed lines indicate the a glide planes perpendicular to the b-axis.

## supplementary crystallographic information

### Comment

As part of our studies on the synthesis and characterization of aroylhydrazone derivatives, we report on the crystal structure of *(E)*-3-hydroxy-*N'*-(2-hydroxybenzylidene)-2-naphthohydrazide.

The asymmetric unit contains one molecule of the title compound, which is shown in Figure 1. The molecule is almost planar with a dihedral angle of 15.27 (13)° between the benzene ring and the naphthyl ring. This configuration is stabilized by two intramolecular hydrogen bonds of the types O–H···N and N–H···O. The keto group acts as an acceptor in a hydrogen bond of the type O–H···O leading to infinite chains along [100] (Fig. 2). In terms of graph-set analysis [Bernstein *et al.* (1995), Etter *et al.* (1990)], the descriptor on the unitary level is C_1_^1^(6)S(6)S(6).

The arrangement of the molecules within the chains along [100] formed by hydrogen bonds becomes evident when viewing along the chain axis (Fig. 3). The chains are constituted by the glide planes perpendicular to the *b*-axis with the glide vectors along [100]. Since the title compound is not oriented parallel or perpendicular to the glide plane, the hydrogen-bond linked molecules do not form layers as one might think when seeing Fig. 2. The repeating unit of the chain consists of two molecules which are oriented approximately perpendicular to each other. The least-square planes (determined by all non-hydrogen atoms of a molecule) of adjacent molecules enclose an angle of 86.33 (1)°.

The same hydrogen bonds as described above are present in the structure of an ethoxy derivative of the title compound [Lei *et al.* (2008)], however, slightly undulated layers are formed. Stronger undulation of the layers is observed in the structure of a methyl derivative of the title compound [Liu *et al.* (2004)], in which the keto group is not involved as acceptor in the intramolecular hydrogen bond, but the hydroxyl group bound to the phenyl ring.

### Experimental

All reagents were commercially available and used as received. A methanol (10 ml) solution of 2-hydroxybenzaldehyde (1.63 mmol) was drop-wise added to a methanol solution (10 ml) of 3-hydroxy-2-naphthohydrazide (1.63 mmol), and the mixture was refluxed for 3 h. Then the solution was evaporated on a steam bath to 5 cm^3^ and cooled to room temperature. Yellow precipitates of the title compound were separated and filtered off, washed with 5 ml of cooled methanol and then dried in air. X-ray quality crystals of the title compound were obtained from methanol by slow solvent evaporation. Yield: 75%, mp 317 °C.

### Refinement

O– and N-bound H atoms were refined freely. C-bonded H atoms were positioned geometrically (C—H = 0.95 Å) and treated as riding on their parent atoms [*U*_iso_(H) = 1.2*U*_eq_(C)].

1550 Friedel pairs have been merged. The Flack parameter is meaningless.

### Crystal data

**Table:**

C_18_H_14_N_2_O_3_	*F*(000) = 640
*M**_r_* = 306.32	*D*_x_ = 1.422 (1) Mg m^−^^3^
Orthorhombic, *P**n**a*2_1_	Mo *K*α radiation, λ = 0.71073 Å
Hall symbol: P 2c -2n	Cell parameters from 5508 reflections
*a* = 12.6749 (4) Å	θ = 3.1–27.5°
*b* = 4.9666 (1) Å	µ = 0.10 mm^−^^1^
*c* = 22.7299 (6) Å	*T* = 200 K
*V* = 1430.87 (7) Å^3^	Rod, yellow
*Z* = 4	0.50 × 0.10 × 0.09 mm

### Data collection

**Table:**

Nonius KappaCCD diffractometer	1416 reflections with *I* > 2σ(*I*)
Radiation source: rotating anode	*R*_int_ = 0.054
MONTEL, graded multilayered X-ray optics	θ_max_ = 27.4°, θ_min_ = 3.3°
Detector resolution: 9 pixels mm^-1^	*h* = −16→16
CCD; rotation images; thick slices, phi/ω–scan	*k* = −6→6
10328 measured reflections	*l* = −29→29
1676 independent reflections	

### Refinement

**Table:**

Refinement on *F*^2^	Primary atom site location: structure-invariant direct methods
Least-squares matrix: full	Secondary atom site location: difference Fourier map
*R*[*F*^2^ > 2σ(*F*^2^)] = 0.036	Hydrogen site location: inferred from neighbouring sites
*wR*(*F*^2^) = 0.095	H atoms treated by a mixture of independent and constrained refinement
*S* = 1.09	*w* = 1/[σ^2^(*F*_o_^2^) + (0.052*P*)^2^ + 0.1709*P*] where *P* = (*F*_o_^2^ + 2*F*_c_^2^)/3
1676 reflections	(Δ/σ)_max_ < 0.001
220 parameters	Δρ_max_ = 0.16 e Å^−^^3^
1 restraint	Δρ_min_ = −0.17 e Å^−^^3^

### Special details

**Table:**

Geometry. All e.s.d.'s (except the e.s.d. in the dihedral angle between two l.s. planes) are estimated using the full covariance matrix. The cell e.s.d.'s are taken into account individually in the estimation of e.s.d.'s in distances, angles and torsion angles; correlations between e.s.d.'s in cell parameters are only used when they are defined by crystal symmetry. An approximate (isotropic) treatment of cell e.s.d.'s is used for estimating e.s.d.'s involving l.s. planes.
Refinement. Refinement of *F*^2^ against ALL reflections. The weighted *R*-factor *wR* and goodness of fit *S* are based on *F*^2^, conventional *R*-factors *R* are based on *F*, with *F* set to zero for negative *F*^2^. The threshold expression of *F*^2^ > 2*σ*(*F*^2^) is used only for calculating *R*-factors(gt) *etc*. and is not relevant to the choice of reflections for refinement. *R*-factors based on *F*^2^ are statistically about twice as large as those based on *F*, and *R*-factors based on all data will be even larger.

### Fractional atomic coordinates and isotropic or equivalent isotropic displacement parameters (Å^2^)

**Table:**

	*x*	*y*	*z*	*U*_iso_*/*U*_eq_	
O1	0.23226 (13)	0.2694 (4)	0.22862 (9)	0.0383 (4)	
O2	0.31563 (16)	−0.3045 (5)	0.09755 (11)	0.0533 (6)	
H2	0.280 (4)	−0.182 (9)	0.124 (2)	0.092 (14)*	
O3	−0.08649 (14)	0.4024 (3)	0.18929 (8)	0.0325 (4)	
H3	−0.151 (3)	0.377 (6)	0.1953 (15)	0.044 (8)*	
N1	0.09013 (15)	0.1311 (4)	0.17680 (9)	0.0283 (4)	
H1	0.019 (2)	0.157 (5)	0.1723 (13)	0.037 (7)*	
N2	0.14658 (15)	−0.0532 (4)	0.14425 (8)	0.0290 (4)	
C1	0.13752 (17)	0.2878 (5)	0.21681 (10)	0.0258 (5)	
C2	0.06987 (17)	0.4915 (5)	0.24682 (10)	0.0250 (5)	
C3	−0.03790 (17)	0.5489 (5)	0.23284 (10)	0.0255 (5)	
C4	−0.09057 (18)	0.7497 (5)	0.26178 (11)	0.0283 (5)	
H4	−0.1612	0.7897	0.2509	0.034*	
C5	−0.04258 (17)	0.8986 (5)	0.30734 (10)	0.0272 (5)	
C6	−0.0956 (2)	1.1070 (5)	0.33832 (11)	0.0332 (6)	
H6	−0.1660	1.1524	0.3280	0.040*	
C7	−0.0461 (2)	1.2432 (5)	0.38286 (11)	0.0353 (6)	
H7	−0.0828	1.3807	0.4035	0.042*	
C8	0.0589 (2)	1.1808 (6)	0.39843 (12)	0.0380 (6)	
H8	0.0925	1.2761	0.4294	0.046*	
C9	0.1119 (2)	0.9845 (5)	0.36906 (11)	0.0344 (6)	
H9	0.1827	0.9443	0.3797	0.041*	
C10	0.06327 (18)	0.8391 (5)	0.32300 (10)	0.0279 (5)	
C11	0.11661 (18)	0.6368 (5)	0.29118 (11)	0.0282 (5)	
H11	0.1880	0.5994	0.3008	0.034*	
C12	0.0899 (2)	−0.1961 (5)	0.10947 (11)	0.0301 (5)	
H12	0.0156	−0.1697	0.1093	0.036*	
C13	0.1346 (2)	−0.3955 (5)	0.07051 (10)	0.0310 (5)	
C14	0.2435 (2)	−0.4434 (6)	0.06574 (12)	0.0372 (6)	
C15	0.2796 (2)	−0.6364 (7)	0.02599 (16)	0.0501 (7)	
H15	0.3532	−0.6656	0.0216	0.060*	
C16	0.2097 (3)	−0.7845 (6)	−0.00686 (14)	0.0488 (8)	
H16	0.2356	−0.9187	−0.0329	0.059*	
C17	0.1027 (3)	−0.7416 (5)	−0.00265 (13)	0.0450 (7)	
H17	0.0548	−0.8429	−0.0259	0.054*	
C18	0.0665 (2)	−0.5492 (5)	0.03591 (11)	0.0388 (6)	
H18	−0.0073	−0.5201	0.0391	0.047*	

### Atomic displacement parameters (Å^2^)

**Table:**

	*U*^11^	*U*^22^	*U*^33^	*U*^12^	*U*^13^	*U*^23^
O1	0.0226 (8)	0.0433 (10)	0.0489 (10)	0.0037 (7)	−0.0027 (8)	−0.0084 (9)
O2	0.0286 (10)	0.0615 (14)	0.0697 (15)	0.0054 (9)	−0.0018 (10)	−0.0210 (12)
O3	0.0207 (8)	0.0394 (10)	0.0373 (10)	−0.0008 (7)	−0.0018 (7)	−0.0050 (8)
N1	0.0209 (9)	0.0316 (10)	0.0324 (10)	0.0024 (8)	0.0015 (8)	−0.0025 (8)
N2	0.0265 (9)	0.0297 (10)	0.0309 (10)	0.0024 (8)	0.0038 (8)	0.0004 (8)
C1	0.0216 (10)	0.0267 (11)	0.0290 (12)	0.0000 (9)	0.0025 (9)	0.0035 (10)
C2	0.0218 (11)	0.0243 (12)	0.0288 (11)	−0.0016 (9)	0.0026 (9)	0.0038 (9)
C3	0.0228 (11)	0.0268 (11)	0.0270 (11)	−0.0034 (9)	−0.0004 (9)	0.0021 (10)
C4	0.0213 (10)	0.0295 (12)	0.0340 (12)	0.0016 (9)	0.0003 (9)	0.0056 (10)
C5	0.0266 (11)	0.0260 (12)	0.0292 (11)	−0.0020 (9)	0.0012 (9)	0.0044 (9)
C6	0.0321 (12)	0.0314 (13)	0.0362 (14)	0.0010 (10)	0.0045 (10)	0.0029 (11)
C7	0.0439 (14)	0.0290 (13)	0.0330 (13)	0.0009 (11)	0.0056 (11)	−0.0026 (11)
C8	0.0447 (15)	0.0367 (14)	0.0326 (13)	−0.0053 (12)	−0.0032 (12)	−0.0016 (11)
C9	0.0341 (12)	0.0335 (13)	0.0357 (13)	−0.0029 (10)	−0.0065 (11)	0.0020 (11)
C10	0.0289 (12)	0.0263 (12)	0.0285 (12)	−0.0030 (10)	−0.0008 (10)	0.0036 (10)
C11	0.0238 (11)	0.0293 (12)	0.0315 (12)	−0.0007 (9)	−0.0016 (9)	0.0023 (10)
C12	0.0270 (11)	0.0311 (13)	0.0320 (11)	0.0009 (10)	0.0028 (10)	0.0020 (11)
C13	0.0338 (12)	0.0287 (13)	0.0305 (13)	0.0016 (9)	0.0028 (10)	0.0023 (10)
C14	0.0335 (12)	0.0361 (15)	0.0419 (14)	0.0007 (10)	0.0018 (11)	−0.0029 (12)
C15	0.0440 (16)	0.0484 (17)	0.0577 (17)	0.0120 (13)	0.0099 (15)	−0.0050 (15)
C16	0.072 (2)	0.0361 (15)	0.0389 (15)	0.0052 (14)	0.0104 (14)	−0.0061 (13)
C17	0.062 (2)	0.0370 (15)	0.0365 (15)	−0.0039 (14)	−0.0021 (13)	−0.0023 (13)
C18	0.0411 (14)	0.0380 (15)	0.0372 (13)	−0.0031 (11)	−0.0018 (12)	−0.0019 (12)

### Geometric parameters (Å, °)

**Table:**

O1—C1	1.234 (3)	C7—H7	0.9500
O2—C14	1.354 (3)	C8—C9	1.360 (4)
O2—H2	0.96 (5)	C8—H8	0.9500
O3—C3	1.374 (3)	C9—C10	1.413 (3)
O3—H3	0.84 (4)	C9—H9	0.9500
N1—C1	1.339 (3)	C10—C11	1.411 (3)
N1—N2	1.378 (3)	C11—H11	0.9500
N1—H1	0.92 (3)	C12—C13	1.444 (3)
N2—C12	1.283 (3)	C12—H12	0.9500
C1—C2	1.491 (3)	C13—C18	1.395 (3)
C2—C11	1.374 (3)	C13—C14	1.405 (3)
C2—C3	1.431 (3)	C14—C15	1.394 (4)
C3—C4	1.368 (3)	C15—C16	1.372 (5)
C4—C5	1.410 (3)	C15—H15	0.9500
C4—H4	0.9500	C16—C17	1.377 (5)
C5—C10	1.419 (3)	C16—H16	0.9500
C5—C6	1.421 (3)	C17—C18	1.375 (4)
C6—C7	1.369 (4)	C17—H17	0.9500
C6—H6	0.9500	C18—H18	0.9500
C7—C8	1.411 (4)		
			
C14—O2—H2	110 (3)	C8—C9—C10	121.0 (2)
C3—O3—H3	114 (2)	C8—C9—H9	119.5
C1—N1—N2	121.20 (19)	C10—C9—H9	119.5
C1—N1—H1	115.9 (18)	C11—C10—C9	122.3 (2)
N2—N1—H1	122.9 (18)	C11—C10—C5	118.2 (2)
C12—N2—N1	114.05 (19)	C9—C10—C5	119.5 (2)
O1—C1—N1	122.8 (2)	C2—C11—C10	122.9 (2)
O1—C1—C2	120.7 (2)	C2—C11—H11	118.5
N1—C1—C2	116.55 (19)	C10—C11—H11	118.5
C11—C2—C3	118.0 (2)	N2—C12—C13	122.5 (2)
C11—C2—C1	116.35 (19)	N2—C12—H12	118.8
C3—C2—C1	125.6 (2)	C13—C12—H12	118.8
C4—C3—O3	120.88 (19)	C18—C13—C14	118.1 (2)
C4—C3—C2	120.3 (2)	C18—C13—C12	118.6 (2)
O3—C3—C2	118.84 (19)	C14—C13—C12	123.3 (2)
C3—C4—C5	121.6 (2)	O2—C14—C15	118.4 (2)
C3—C4—H4	119.2	O2—C14—C13	122.4 (2)
C5—C4—H4	119.2	C15—C14—C13	119.2 (3)
C4—C5—C10	118.9 (2)	C16—C15—C14	120.7 (3)
C4—C5—C6	122.8 (2)	C16—C15—H15	119.7
C10—C5—C6	118.3 (2)	C14—C15—H15	119.7
C7—C6—C5	120.7 (2)	C15—C16—C17	121.0 (3)
C7—C6—H6	119.7	C15—C16—H16	119.5
C5—C6—H6	119.7	C17—C16—H16	119.5
C6—C7—C8	120.6 (2)	C18—C17—C16	118.7 (3)
C6—C7—H7	119.7	C18—C17—H17	120.6
C8—C7—H7	119.7	C16—C17—H17	120.6
C9—C8—C7	120.0 (2)	C17—C18—C13	122.2 (3)
C9—C8—H8	120.0	C17—C18—H18	118.9
C7—C8—H8	120.0	C13—C18—H18	118.9
			
C1—N1—N2—C12	177.3 (2)	C4—C5—C10—C11	1.4 (3)
N2—N1—C1—O1	−3.1 (3)	C6—C5—C10—C11	−178.4 (2)
N2—N1—C1—C2	176.38 (18)	C4—C5—C10—C9	−179.2 (2)
O1—C1—C2—C11	−5.6 (3)	C6—C5—C10—C9	1.0 (3)
N1—C1—C2—C11	174.9 (2)	C3—C2—C11—C10	−0.3 (3)
O1—C1—C2—C3	173.6 (2)	C1—C2—C11—C10	179.0 (2)
N1—C1—C2—C3	−5.9 (3)	C9—C10—C11—C2	179.1 (2)
C11—C2—C3—C4	2.3 (3)	C5—C10—C11—C2	−1.5 (3)
C1—C2—C3—C4	−177.0 (2)	N1—N2—C12—C13	178.3 (2)
C11—C2—C3—O3	−178.9 (2)	N2—C12—C13—C18	177.4 (2)
C1—C2—C3—O3	1.8 (3)	N2—C12—C13—C14	−2.4 (4)
O3—C3—C4—C5	178.8 (2)	C18—C13—C14—O2	−179.9 (3)
C2—C3—C4—C5	−2.4 (4)	C12—C13—C14—O2	−0.1 (4)
C3—C4—C5—C10	0.5 (3)	C18—C13—C14—C15	1.5 (4)
C3—C4—C5—C6	−179.7 (2)	C12—C13—C14—C15	−178.7 (3)
C4—C5—C6—C7	179.0 (2)	O2—C14—C15—C16	179.3 (3)
C10—C5—C6—C7	−1.2 (3)	C13—C14—C15—C16	−2.1 (5)
C5—C6—C7—C8	0.7 (4)	C14—C15—C16—C17	1.8 (5)
C6—C7—C8—C9	0.1 (4)	C15—C16—C17—C18	−0.9 (4)
C7—C8—C9—C10	−0.3 (4)	C16—C17—C18—C13	0.4 (4)
C8—C9—C10—C11	179.1 (2)	C14—C13—C18—C17	−0.7 (4)
C8—C9—C10—C5	−0.2 (4)	C12—C13—C18—C17	179.5 (2)

### Hydrogen-bond geometry (Å, °)

**Table:**

*D*—H···*A*	*D*—H	H···*A*	*D*···*A*	*D*—H···*A*
O2—H2···N2	0.96 (5)	1.87 (5)	2.697 (3)	142 (4)
O3—H3···O1^i^	0.84 (4)	1.81 (4)	2.609 (2)	157 (3)
N1—H1···O3	0.92 (3)	1.85 (3)	2.628 (3)	141 (2)

Symmetry codes: (i) *x*−1/2, −*y*+1/2, *z*.
